# The Book World of Medicine and Science

**Published:** 1894-07-14

**Authors:** 


### The Book World of Medicine and Science.

**Willing's British and Irish Press Guide and Advertiser's Directory and Handbook for 1894.** Price 1s. (J. Willing, 125, Strand.)

We have received the "Press Guide" for 1894, this being its twenty-first year of publication. Into a small space much useful information is condensed, and to busy people the consequent convenience for purposes of reference will be duly appreciated. A full list of newspapers and periodicals occupies 131 pages, and is followed by a classification of subjects, which greatly facilitates the hunting up of special publications. Alterations in titles and so forth are exhaustively dealt with, and continental and foreign newspapers receive due share of attention. On page 224 some interesting information as to newspapers and periodicals of the seventeenth and eighteenth centuries is given. The book is certainly a wonderful shillingsworth.

**Health Resorts of Northumberland and Durham, &c.** By Richard Ellis, F.R.G.S., with Introduction by William Murray, M.D. (London: Walter Scott, Limited. 1894.)

Dr. Ellis gives in this small volume very interesting general information about a part of our island of which not much is known by the average Englishman, though the northern counties of Northumberland and Durham are as rich in historical association and beautiful scenery as any corner of the British Isles. Dr. Ellis's special object is to point out how very many places there are, both inland and on the coast, suitable for invalids whose homes are in the North, and who may be thankful to be spared the fatigue and expense of the journey South. The description of Lindisfarne, "Cuthbert's islet grey," with its splendid air, the best substitute for a sea voyage to be had, its grand old ruined abbey and its ancient church, with their countless historical memories, makes the reader long for an opportunity to visit "Holy Island." Many particulars are given of some forty or fifty resorts, pointing out the class of cases suitable for each. Bamborough, Newbiggen, Tynemouth, for instance, are recommended for those suffering from nervous debility, rheumatism, and all scrofulous complaints, the season being from July to October. For chronic rheumatism and rheumatoid arthritis, the sulphur pools at Croft, locally known as "Hell Kettles," have been found to work wonders, as also in cases of phthisis. We warmly recommend the study of Dr. Ellis's useful and interesting book to anyone contemplating a stay in Northern England. It is a wonderful sixpennyworth of information. A chapter on "Hints on Sea Bathing" is added by Professor Philipson, M.D.

**Nature, Wild Sport, and Humble Life.** By Aubyn Trevor-Battye, B.A. (London: Longman, Green, and Co.)

This is an interesting little volume containing some eight or nine short stories told by an amateur naturalist as he walks along the by-paths of natural history, always avoiding the cut-and-dried high roads of pure science. His first story is of "Carl of the Hill," who in a great forest in the very heart of Scandinavia combines the occupation of hunter, farmer, timber cutter, and naturalist. The rugged honest nature of this child of the forest, with an eye for the beautiful in nature and a blind devotion for his little girl, is delightfully refreshing, and very pathetic is the story of her death in one of her father's own log traps, which he had set to ensnare the roving elk of the forest. Lovers of Oxford will appreciate the author's interpretation of the "Upper River": "Now you have seen a little of the 'upper river' to-day, but you must not think you know it yet; when you have seen it in the dawn, when you have felt your way at night round every winding reach, and listened to and learnt the meaning of the night voices, then and not till then may you claim to know a little of the upper river." The author has a most happy way of catching the spirit of his surroundings, whether on land, or sea. Fishermen will rejoice in the "Three Fishes," an account of three marvellous catches, but every one will find something to his or her taste, and dumb must be the man in whom some note cannot be raised to sympathetic vibrations by the tuneful and simple harmony of this young naturalist.

